# Development of a rapid quantitative method to differentiate MS1 vaccine strain from wild-type *Mycoplasma synoviae*

**DOI:** 10.3389/fvets.2024.1354548

**Published:** 2024-03-01

**Authors:** Changtao Liao, Yiquan Chen, Zhuanqiang Yan, Yiwei Song, Qi Zhou, Puduo Zhu, Xudong He, Wenyang Li, Feng Chen

**Affiliations:** ^1^College of Animal Science, South China Agricultural University, Guangzhou, China; ^2^Yunfu Branch, Guangdong Laboratory for Lingnan Modern Agriculture, Yunfu, China

**Keywords:** real-time PCR, differentiation methods, *Mycoplasma synoviae*, MS attenuated live vaccines, wild-type strains

## Abstract

*Mycoplasma synoviae* (MS) is an economically important pathogen in the poultry industry. Vaccination is an effective method to prevent and control MS infections. Currently two live attenuated MS vaccines are commercially available, the temperature-sensitive MS-H vaccine strain and the NAD-independent MS1 vaccine strain. Differentiation of vaccine strains from wild-type (WT) strains is crucial for monitoring MS infection, especially after vaccination. In this study, we developed a Taqman duplex real-time polymerase chain reaction (PCR) method to identify MS1 vaccine strains from WT strains. The method was specific and did not cross-react with other avian pathogens. The sensitivity assay indicated that no inhibition occurred between probes or between mixed and pure templates in duplex real-time PCR. Compared with the melt-based mismatch amplification mutation assay (MAMA), our method was more sensitive and rapid. In conclusion, the Taqman duplex real-time PCR method is a useful method for the diagnosis and differentiation of WT-MS and MS1 vaccine strains in a single reaction.

## 1 Introduction

*Mycoplasma synoviae* (MS) has been described as an important pathogen causing air sacculitis, infection synovitis and eggshell apex abnormalities (1–3), and is listed as a notifiable *mycoplasma* by the World Organization for Animal Health (WOAH) (4). MS infection can cause subclinical symptoms and lead to co-infection with *Mycoplasma gallisepticum* (MG), Newcastle disease virus (NDV), Infectious bronchitis virus (IBV), and other avian pathogens (5–8). Rapid and accurate diagnosis is necessary to monitor MS infection especially after vaccination. Diagnostic methods for MS include bacteriological isolation, serological assays and molecular detection (9). *Mycoplasma* isolation is inefficient and expensive, as *in vitro* growth requires a rich medium and is time-consuming (10, 11). The serological assay only provides a history of infection (12). Molecular analysis, such as polymerase chain reaction (PCR) or real-time PCR (qPCR), guarantees earlier detection, is more rapid, more sensitive, and more specific than the others, and is widely used (9).

Measures to prevent and control MS include vaccines and antibiotics. However, the emergence of drug resistance in MS strains has made the use of antibiotics more cautious (13–16). Vaccination is another option to control the disease. At present, in addition to the inactivated vaccine, only two live attenuated vaccines are commercially available: the temperature-sensitive (ts^+^) MS-H vaccine strain (Vaxsafe^®^ MS, Bioproperties Pty Ltd.) and the NAD-independent MS1 vaccine strain (Nobilis^®^ MS Live, MSD Animal Health Inc.). The MS-H strain was developed by chemical mutagenesis of an Australian strain (86079/7NS), while the MS1 strain is a spontaneous attenuation of the wild-type pathogenic isolate WVU1853. After live vaccine inoculation, the differentiation of vaccine strains from wild-type strains is crucial for monitoring MS infection. Moreover, it is important to determine whether the vaccine strains have successfully colonized the respiratory mucosa to provide effective protection against wild-type (WT) strains (17, 18). Several genotyping techniques have been developed to differentiate MS-H strains from WT strains, including real-time PCR (19), melting curve analysis, agarose gel-based mismatch amplification mutation assay (MAMA) (20), and high-resolution melting curve assays (21, 22). However, only one study provided a way to distinguish the MS1 strain from WT strains, using melt-based MAMA PCR or agarose-MAMA PCR (23).

In this study, we developed a Taqman duplex real-time PCR method that was sensitive, specific and more rapid than melt-based MAMA. The developed method is applicable both in laboratory and clinical testing, and promotes an easier method to differentiate WT-MS strains and MS1 vaccine strains in a single reaction.

## 2 Materials and methods

### 2.1 Samples

The MS1 (Nobilis^®^ MS Live, MSD) and MS-H (Vaxsafe^®^ MS-H, SINDER) vaccine strains used in this study were obtained from commercial distributors. The WT- MS strains and DNA samples extracted from tracheal swab samples were isolated by the authors (Supplementary Table S1). The genomes of MG, IBV, NDV, Avian influenza virus (AIV), Avian reovirus (ARV), *Escherichia coli* (*E. coli*) and *Avibacterium paragallinarum* (*A. paragallinarum*) were used for the specific detection of the method. The standard nucleic acid (plasmid) of MS1 and WT- MS used in this study was constructed with pMD-18 T (Takara, China).

### 2.2 Nucleic acid extraction

The nucleic acids of MS, MG, *E.coli* and *A. paragallinarum* were extracted using the Bioer Total DNA Extraction Kit (Bioer Tec., China). The nucleic acids of IBV, NDV, AIV and ARV were extracted using the Bioer Total RNA Extraction Kit (Bioer Tec., China) and then the extracted RNAs were used to synthesize cDNAs using the PrimeScript™ II 1st Strand cDNA Synthesis Kit (Takara, China) according to the manufacturer’s instructions.

### 2.3 Monoplex and duplex real-time PCR

By sequencing and comparing the whole genomes of the vaccine strain and wild-type strains (24), we found a single nucleotide mutation site, and designed probes and primers that could be used to distinguish the vaccine strain (MS1) from wild-type strains (Supplementary Table S2). All real-time PCR reactions were carried out on an ABI 7500fast Real-time PCR Detection System. A volume of 20 μL reaction mixture contained 10 μL 2x THUNDERBIRD Probe qPCR Mix (TOYOBO, China), 200 nM each primer, 100 nM each probe, and 2 μL templates. The reaction conditions involved incubation at 95°C for 3 min, followed by 40 cycles of denaturation at 95°C for 15 s and a combined annealing and extension step at 60°C for 30s.

For the sensitivity of real-time PCR assays, MS1 and MS-WT standard plasmids were constructed. Briefly, qPCR amplification products were collected, purified by gel, and then connected to the pMT-18 T vector. The recombinant plasmids with correct sequencing were used as the standard plasmids for subsequent experiments. Each standard plasmid was serially diluted tenfold to achieve concentrations of 10^1^ to 10^7^ copies/μL. The serially diluted plasmids were used to establish a standard curve for each target after three technical replications. For duplex real-time PCR, two plasmids were equally mixed and then serially diluted as described above. The single and mixed plasmids were used to compare detection sensitivities between the duplex reaction and the individual singular reactions.

For the specificity assay, potential cross-reactions with other avian pathogens were measured to ensure the specificity of our method. The templates used in this assay included DNA from MS1, MS-H, MS-WT, MG, *E.coli* and *A. paragallinarum*, and cDNA from IBV, NDV, AIV, and ARV.

### 2.4 Melt-based mismatch amplification mutation assays

As described by Kreizinger et al. (23), MAMA is based on allele-specific competing primers and is widely used for SNP detection. One volume of Melt-MAMA PCR reaction was performed in 20 μL, containing 2 μL templates, 150 nM each primer, 4 μL 5x Colorless GoTaq Flexi Buffer (Promega), 2 μL MgCl_2_ (25 mM), 0.6 μL dNTP (10 mM, Takara), 1 μL EvaGreen (Biotium Inc.) and 0.16 μL GoTaq DNA polymerase (5 U/μL, Promega). Melt-based MAMA PCR reactions were carried out on an ABI 7500fast Real-time PCR Detection System with High Resolution Melting (HRM) Software (v3.2, Thermo Fisher). The thermocycling parameters were 95°C for 10 min, followed by 40 cycles of 95°C for 15 s and 58°C for 1 min and a dissociation protocol comprising 95°C for 15 s, followed by an incremental temperature ramp (0.2°C) from 58°C to 95°C.

### 2.5 Image and statistical analyses

All graphs and statistics in this study were created with GraphPad Prism 8 software (v8.0.2). All data are presented as standard errors (SEs) of at least three independent experiments.

## 3 Results

### 3.1 Duplex real-time PCR specificity analysis

To determine the specificity of the method, genomes extracted from different chicken pathogens were used as templates. As shown in Table 1, no specific amplifications occurred between the reaction systems of other avian pathogens. As expected, in the duplex real-time PCR reaction system, the nucleic acids of WT-MS were positive in the MS-WT measurement channel and negative in the MS1 measurement channel and vice versa. The MS-H strain was identified as the WT strain in the duplex real-time PCR system.

**Table 1:** Specificity of the duplex real-time polymerase chain reaction (PCR).

Pathogens	Nucleic acid type	MS-WT channel (Ct)	MS1 channel (Ct)	Detection results
MS-WT	DNA	21.0365	/	Wild-type positive
MS1	DNA	/	19.2354	MS1 vaccine positive
MS-WT + MS1	DNA	21.8562	20.0004	Both wild-type and MS1 vaccine positive
MS-H	DNA	19.2650	/	Wild-type positive
MG	DNA	/	/	Negative
IBV	cDNA	/	/	Negative
NDV	cDNA	/	/	Negative
AIV	cDNA	/	/	Negative
ARV	cDNA	/	/	Negative
*E.coli*	DNA	/	/	Negative
*A. paragallinarum*	DNA	/	/	Negative

### 3.2 Duplex real-time PCR sensitivity analysis

The sensitivity of the duplex real-time PCR was investigated from two perspectives. One was to compare the detection limits of monoplex qPCR with duplex qPCR. The other was carried out with templates containing a single target or mixed targets.

**Figure 1:**
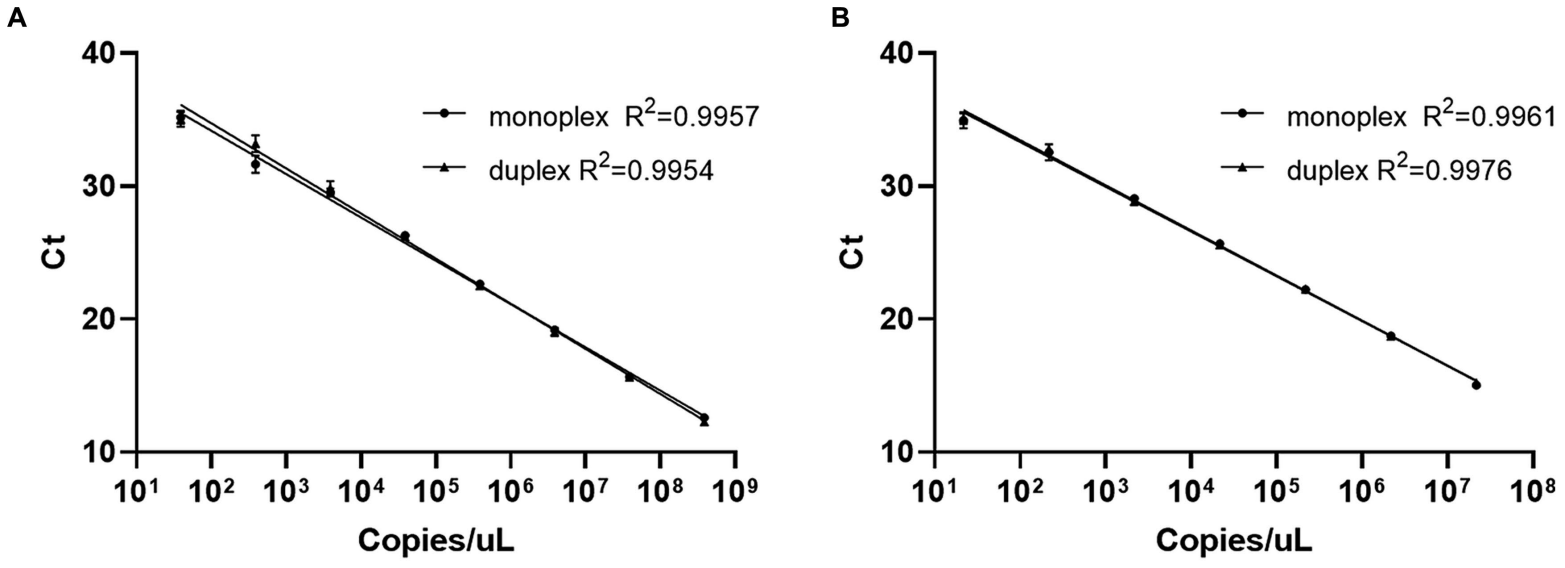
Standard curves of monoplex and duplex real-time PCRs with the same targets. **(A)** Standard curves of monoplex and duplex real-time PCRs using the MS1 standard plasmid template. **(B)** Standard curves of monoplex and duplex real-time PCRs using the MS-WT standard plasmid template.

To make the comparison more intuitive, we constructed the standard plasmids of MS-WT and MS1 respectively, and plotted the standard curves. As shown in Figure 1, targeting the same plasmids, the curves shown for duplex and monoplex real-time PCRs, respectively, were in practical agreement, and the minimum detection limits of all qPCRs were between 10^1^ and 10^2^ copies/μL (Figure 1). No evidence of inhibition between probes was observed in the duplex reaction.

Do mixed targets have any effect on duplex real-time PCR? The assay was performed with single or mixed plasmids of MS-WT and MS1. As shown in Table 2, similar Ct values were obtained from the same target in mixed and single templates. There was no evidence of inhibition as both targets only reacted with their specific probe.

**Table 2:** Sensitivity of the duplex real-time polymerase chain reaction (PCR).

Templates^a^	Duplex real-time PCR, MS-WT channel		Duplex real-time PCR, MS1 channel	
	Mean Ct	SE^b^	Mean Ct	SE^b^
MS-WT-P1	22.0793	0.1097	/	/
MS-WT-P2	25.5165	0.1283	/	/
MS-WT-P3	29.1750	0.0075	/	/
MS-WT-P4	32.7358	0.1617	/	/
MS1-P1	/	/	19.3164	0.1655
MS1-P2	/	/	23.0032	0.1855
MS1-P3	/	/	26.9095	0.1806
MS1-P4	/	/	29.9573	0.1378
MS1-P1 + WT-P1	22.1378	0.1732	19.7309	0.1414
MS1-P2 + WT-P2	25.9738	0.0181	23.6055	0.1411
MS1-P3 + WT-P3	29.5684	0.1176	27.3696	0.1996
MS1-P4 + WT-P4	32.9125	0.0226	30.4886	0.0219

a *p* refers to the plasmid, the number represents different dilution multiples of the plasmid.

b SE indicates standard error.

### 3.3 Comparison of duplex real-time PCR with melt-based MAMA

To confirm the practicality of duplex real-time PCR, we detected the same templates by real-time PCR and melt-based MAMA methods, respectively. The template information is shown in Table 1. Results showed that both methods could distinguish between WT strains and the MS1 vaccine strain (Table 3). Test results of clinical swab samples showed a higher detection rate of duplex real-time PCR than melt-based MAMA, especially when the nucleic acid content of the samples was low (Supplementary Table S3). It should be noted that the melt-based MAMA method was more suitable for qualitative analysis because the instrumentation system only showed the Ct values of the higher peak when there were two detection targets in the same sample (Table 3). All of the results indicated that the developed duplex real-time PCR method was more sensitive and more suitable for quantitative analysis than the existing method.

**Table 3:** Ct values of samples detected by duplex real-time polymerase chain reaction (PCR) and melt-based mismatch amplification mutation assay (MAMA) method.

Samples	Duplex real-time PCR		Melt-based MAMA	
	MS-WT channel (Ct)	MS1 channel (Ct)	MS-WT channel^a^ (Ct)	MS1 channel^a^ (Ct)
MS1	/	25.13	/	30.93
MS-WT1	18.6	/	23.75	/
MS-WT2	26.27	/	31.97	/
MS-WT3	22.76	/	28.21	/
SI-1	26.40	/	32.51	/
SI-2	28.27	/	34.08	/
SI-3	26.71	/	33.09	/
SI-7	32.85	/	/	/
SV-7	33.18	29.15	/	34.59
SV-8	33.50	28.23	B^b^	33.98
SV-9	31.84	29.17	/	35.07
SV-10	28.20	30.28	B^b^	36.40

a The MS1 and MS-WT channels represent the melting temperatures of the MS1 vaccine strain (approximately 75°C) and wild-type strains (approximately 70°C) respectively.

b B indicates that there is a peak near the corresponding Tm value, but no Ct value.

In addition, the duplex real-time PCR took less time for detection than the existing method because there was no slow warming step (0.2°C/s).

## 4 Discussion

MS is distributed worldwide and has become one of the most important pathogens threatening the global poultry industry (24, 25). Furthermore, MS co-infections with other infectious agents such as NDV, IBV, *E. coli*, and MG increase economic losses (26–28). Research on MS can lay the foundation for the prevention and treatment of MS-related diseases. With the increase in positive and incidence rates, prevention and control of MS have become the focus of the poultry industry in China (29–31).

To increase the knowledge of MS epidemiology and to improve control and eradication programs, it is important to monitor MS infection and identify sources of infection and modes of transmission. In order to avoid economic losses due to disease outbreaks, vaccination has become the primary prevention and control measure in the poultry industry. For MS, the live vaccine stands out among other types of vaccines because it prevents infection with wild-type strains by colonizing the trachea and continuously stimulating the immune response (17, 18). To date, only two commercial live vaccines are available in the world. After immunization with a live vaccine, differentiation between wild-type strains and vaccine strains is imperative. There have been several reports on distinguishing the MS-H vaccine strain from wild-type strains but only one report on the MS1 vaccine strain (23). In this study, we developed a quantitative and rapid Taqman-based duplex real-time PCR method to differentiate and quantify the MS1vaccine strain and wild-type strains simultaneously.

The specificity assay indicated that no fluorescent signal was detected among the nucleic acids of MG, IBV, NDV, AIV, ARV, *E. coli* and *A. paragallinarum* in our reaction system. Since the purpose of this study is to distinguish the MS1 strain from the wild-type strains, we did not take the MS-H strain into account. According to the results, the MS-H strain was identified as wild-type as expected. The quantification method requires knowledge of the detection limit. Therefore, we determined the limit of our method by 10-fold serial dilution of the standard plasmids. The lowest detection range was between 10^1^ and 10^2^ copies/μL, regardless of whether it was an MS1 plasmid or MS-WT plasmid. We also found that duplex reactions or mixed targets did not significantly influence the detection results.

Duplex real-time PCR has distinct advantages over melt-based MAMA, because it allows quantification and differentiation at the same time. In addition, the detection limit of duplex real-time PCR is more sensitive than that of melt-based MAMA considering the lower Ct values.

## 5 Conclusion

In conclusion, a duplex real-time PCR method was developed to distinguish between wild-type MS strains and MS1 vaccine strains. This method was highly specific and sensitive, and allowed the simultaneous quantification of MS1 and MS-WT. Based on the above, duplex real-time PCR can be used as a diagnostic tool for the detection and quantification of MS strains after inoculation with the MS1 live vaccine.

## Data availability statement

The original contributions presented in the study are included in the article/Supplementary material, further inquiries can be directed to the corresponding authors.

## Author contributions

CL: Writing – original draft. YC: Writing – review & editing. ZY: Investigation, Writing – review & editing. YS: Methodology, Writing – review & editing. QZ: Writing – review & editing, Software. PZ: Writing – review & editing, Data curation. XH: Writing – review & editing, Formal analysis. WL: Writing – review & editing, Resources. FC: Writing – review & editing, Investigation.
