# Supplementary material for: Development of a rapid quantitative method to differentiate MS1 vaccine strain from wild-type Mycoplasma synoviae
Source: Front Vet Sci. 2024 Mar 1;11:1354548. doi: 10.3389/fvets.2024.1354548 (PMC10940412; doi:10.3389/fvets.2024.1354548)
Supplement: Supplementary file 1 [file Table_1.DOCX]

**Supplementary table 1.** Background information and genotype of the *Mycoplasma synoviae* strains and clinical samples included in this study.

| **Sample ID** | **Sample type^a^** | **Host** | **Age of host (weeks)** | **Type of host** | **Origin of sample^b^** | **Year** | **Gt^c^** |
| --- | --- | --- | --- | --- | --- | --- | --- |
| MS1 | MS1 vaccine strain |  |  |  | Nobilis® MS Live, MSD Animal Health |  | MS1 |
| MS-H | ts+ MS-H vaccine strain |  |  |  | Vaxsafe® MS, Bioproperties Pty Ltd. |  | WT |
| MS-WT1 | *Mycoplasma synoviae* strains | chicken | 37 | broiler | farm 3 GD-XX China | 2016 | WT |
| MS-WT2 | *Mycoplasma synoviae* strains | chicken | 65 | broiler | farm 4 GD-XX China | 2018 | WT |
| MS-WT3 | *Mycoplasma synoviae* strains | chicken | 72 | broiler | farm 8 JS-GY China | 2016 | WT |
| MS-WT4 | *Mycoplasma synoviae* strains | chicken | 38 | broiler | farm 2 SD-TA China | 2017 | WT |
| MS-WT5 | *Mycoplasma synoviae* strains | chicken | 45 | broiler | farm 3 GX-YL China | 2019 | WT |
| MS-WT6 | *Mycoplasma synoviae* strains | chicken | 50 | broiler | farm 5 SC-DY China | 2020 | WT |
| MS-WT7 | *Mycoplasma synoviae* strains | chicken | 53 | broiler | farm 1 YG-GZ China | 2016 | WT |
| MS-WT8 | *Mycoplasma synoviae* strains | chicken | 35 | broiler | farm 3 HN-HB China | 2018 | WT |
| MS-WT9 | *Mycoplasma synoviae* strains | chicken | 69 | broiler | farm 6 SD-DZ China | 2019 | WT |
| MS-WT10 | *Mycoplasma synoviae* strains | chicken | 70 | broiler | farm 4 GD-QY China | 2021 | WT |
| SI-1 | swabs from infected animals | chicken | 3 | broiler | farm 4 GD-XX China | 2019 | WT |
| SI-2 | swabs from infected animals | chicken | 9 | broiler | farm 1 GD-QY China | 2018 | WT |
| SI-3 | swabs from infected animals | chicken | 25 | broiler | farm 4 FJ-XM China | 2016 | WT |
| SI-4 | swabs from infected animals | chicken | 16 | broiler | farm 7 HB-XY China | 2021 | WT |
| SI-5 | swabs from infected animals | chicken | 21 | broiler | farm 3 HB-HD China | 2017 | WT |
| SI-6 | swabs from infected animals | chicken | 8 | breeder | farm 9 GD-XX China | 2021 | WT |
| SI-7 | swabs from infected animals | chicken | 6 | breeder | farm 4 GD-JR China | 2020 | WT |
| SI-8 | swabs from infected animals | chicken | 24 | breeder | farm 1 LH-ZX China | 2016 | WT |
| SI-9 | swabs from infected animals | chicken | 40 | breeder | farm 8 GX-YL China | 2019 | WT |
| SI-10 | swabs from infected animals | chicken | 68 | breeder | farm 2 GX-JM China | 2020 | WT |
| SV-1 | swabs from vaccinated animals | chicken | 4 | breeder | farm 3 ZJ-HZ China | 2018 | MS1 |
| SV-2 | swabs from vaccinated animals | chicken | 6 | breeder | farm 7 JS-CZ China | 2021 | MS1 |
| SV-3 | swabs from vaccinated animals | chicken | 10 | breeder | farm 5 AH-LA China | 2016 | MS1 |
| SV-4 | swabs from vaccinated animals | chicken | 15 | breeder | farm 4 GJ-CZ China | 2017 | MS1 |
| SV-5 | swabs from vaccinated animals | chicken | 17 | breeder | farm1 GD-JM China | 2021 | MS1 |
| SV-6 | swabs from vaccinated animals | chicken | 20 | breeder | farm 5 ZJ-XH China | 2018 | MS1 |
| SV-7 | swabs from vaccinated animals | chicken | 23 | breeder | farm 6 AH-HF China | 2021 | MS1+WT |
| SV-8 | swabs from vaccinated animals | chicken | 27 | breeder | farm 3 GX-HK China | 2020 | MS1+WT |
| SV-9 | swabs from vaccinated animals | chicken | 30 | breeder | farm 4 HN-CS China | 2018 | MS1+WT |
| SV-10 | swabs from vaccinated animals | chicken | 43 | breeder | farm 1 GD-HZ China | 2019 | MS1+WT |

^a^ Swabs were collected form cleft palate of chickens.

^b^ Farm, region and country of origin of the samples.

^c^ Gt: genotype WT: wild-type, MS1: MS1 vaccine.
