# Supplementary material for: Development of a rapid quantitative method to differentiate MS1 vaccine strain from wild-type Mycoplasma synoviae
Source: Front Vet Sci. 2024 Mar 1;11:1354548. doi: 10.3389/fvets.2024.1354548 (PMC10940412; doi:10.3389/fvets.2024.1354548)
Supplement: Supplementary file 2 [file Table_2.DOCX]

**Supplementary table 2.** Primers and probes used to distinguish vaccine strain (MS1) from wild-type strains.

| Primer | Sequence |
| --- | --- |
| MS-F | GAGGAACCGATATTAAGCCATCA |
| MS-R | TTGACGTCCTGAACGACCTCTT |
| Probe-WT-FAM | CTCGGCGGACTAT |
| Probe-MS1-CY5 | CTCGGCGGATTAT |
