# Supplementary material for: Development of a rapid quantitative method to differentiate MS1 vaccine strain from wild-type Mycoplasma synoviae
Source: Front Vet Sci. 2024 Mar 1;11:1354548. doi: 10.3389/fvets.2024.1354548 (PMC10940412; doi:10.3389/fvets.2024.1354548)
Supplement: Supplementary file 3 [file Table_3.DOCX]

**Supplementary table 3.** Overall results of the duplex real-time polymerase chain reaction (PCR) compared with melt-based mismatch amplification mutation assay (MAMA).

| **Sample type** | **Sample size** | **Duplex real-time PCR** | | | **Melt-based MAMA** | | |
| --- | --- | --- | --- | --- | --- | --- | --- |
|  |  | **MS-WT**  **positive** | **MS1**  **positive** | **negative** | **MS-WT**  **positive** | **MS1**  **positive** | **negative** |
| Mycoplasma synoviae  strains | 12 | 11 | 1 | 0 | 11 | 1 | 0 |
| Swabs from infected  animals | 10 | 10 | 0 | 0 | 9 | 0 | 1 |
| Swabs from vaccinated animals | 10 | 4 | 10 | 0 | 2 | 10 | 0 |
